# Outcome of a risk-related therapeutic strategy used prospectively in a population-based study of Hodgkin's lymphoma in adolescents

**DOI:** 10.1038/sj.bjc.6603809

**Published:** 2007-05-29

**Authors:** G L Jones, P R A Taylor, K P Windebank, N A Hoye, H Lucraft, K Wood, B Angus, S J Proctor

**Affiliations:** 1 Newcastle upon Tyne NHS Foundation Trust, Newcastle upon Tyne NE1 4LP, UK; 2 Paediatric Oncology, Newcastle upon Tyne NHS Foundation Trust, Newcastle upon Tyne NE1 4LP, UK; 3 Northumbria Healthcare NHS Trust, North Shields Tyne & Wear NE29 8NH, UK; 4 Northern Centre for Cancer Treatment, Newcastle upon Tyne NHS Foundation Trust, Newcastle upon Tyne NE4 6BE, UK; 5 Academic Haematology, Medical School, Newcastle University, Newcastle upon Tyne NE2 4HH, UK

**Keywords:** Hodgkin lymphoma, adolescent, population study, chemotherapy

## Abstract

The aim was to assess outcome in a population-based cohort of adolescents with Hodgkin's lymphoma (HL) diagnosed in the UK's northern region over a 10-year period. Among a population of 3.09 million, 55 of 676 patients (8%) diagnosed with HL were aged 13–19. Seven had nodular lymphocyte-predominant HL, 48 classical HL (cHL). Of the latter, 36 were ⩾16 years. Application of the Scottish and Newcastle Lymphoma Group (SNLG) prognostic index meant 21 patients were considered high risk (index ⩾0.5). They received PVACEBOP multi-agent chemotherapy as primary therapy. Standard risk patients (SNLG index <0.5) were treated with standard ChlVPP or ABVD chemotherapy±radiotherapy. Scottish and Newcastle Lymphoma Group indexing is not valid for patients under 16. Twelve patients therefore received UKCCSG protocols (*n*=8), ABVD plus radiotherapy (*n*=2), or PVACEBOP (*n*=2). Forty-six patients with cHL (96%) achieved complete remission. Seven patients relapsed but all entered complete remission after salvage therapy. Five patients died: three of HL, one in an accident and one of disseminated varicella complicating cystic fibrosis. Five- and 10-year overall survival was 93 and 86%, respectively; disease-specific survival was 95 and 92%. The data suggest that older adolescents with high-risk HL require intensive protocols as primary therapy to secure optimal outcome.

Hodgkin's lymphoma (HL) is a rare malignancy, with an incidence of approximately 2.4 per 100 000 per annum in developed countries. In these countries, the incidence of the disease is very low in young children, peaks in young adults, wanes in middle age then gradually increases again over the later decades (Kennedy *et al*, 1985; Armstrong *et al*, 1994). During the teenage years there is a steep rise in the incidence of HL, culminating in the peak incidence described in the third decade. There is increasing circumstantial evidence that HL in young children may be the result of a different pathological process to that occurring in young adults.

One potentially significant observation is that HL in young children is more clearly Epstein–Barr virus (EBV) associated than in young adults. This may be important as there is increasing evidence to suggest that the EBV status of tumour cells in HL has an impact on prognosis. This effect may, however, be age dependent (Jarrett *et al*, 2005; Keegan *et al*, 2005), although these observations have not been replicated in all studies (Jarrett *et al*, 2005).

Differences in histological subtype of HL in different age cohorts may also be important. While in children, as in adults, the nodular sclerosing (NS) subtype predominates, up to 30% may have mixed cellularity (MC) disease. Among 17–20 year olds, however, the incidence of MC disease is lower, affecting as few as 4% of individuals in some studies.

Thus, it could be argued that adolescents as a group are susceptible to both young adult-type and childhood-type HL, or alternatively, that they may actually represent a unique cohort with respect to the epidemiology, pathophysiology and potentially prognosis of HL (Windebank, 2005).

In addition to the heterogeneous biological features of HL presenting during the teenage years, it is pertinent to consider the treatments and modes of treatment delivery to this group of patients in the UK. The multidisciplinary team responsible for the management of teenagers with HL varies and is dependent upon local healthcare configurations. In many regions, patients aged >16 years are treated by adult haematologists or oncologists at local hospitals, while those aged <16 years are managed by paediatric oncology teams, generally at regional centres. More recently, in some areas, patients are being treated on designated teenage cancer units either by paediatric or adult teams. It is unsurprising, therefore, that there are considerable variations in the therapeutic regimens used in the management of these patients; while some patients are treated using regimens primarily designed for children (Jenkin *et al*, 1982; Ekert *et al*, 1988; Hudson *et al*, 1993; Hunger *et al*, 1994; Shankar *et al*, 1997, 1998; Weiner *et al*, 1997; Hutchinson *et al*, 1998; Schellong *et al*, 1999; Landmann-Parker *et al*, 2000), others are managed using adult-type approaches (Proctor *et al*, 1991; Yung *et al*, 2004).

Since 1991, patients aged 13–19 years and all adults diagnosed with HL in the Northern Region of England, have been prospectively managed using a risk-adapted treatment approach. The Scotland and Newcastle Lymphoma Group (SNLG) prognostic index (Figure 1A), proven to be of value in identifying high-risk patients >15years old (Proctor *et al*, 1991), has been used to determine whether patients would benefit from intensified treatment. For those too young for valid use of the SNLG index, poor risk status was assigned to those with high-stage and very bulky or extensive extra-nodal disease – factors later confirmed to be of significance by Smith *et al*, 2003 (Figure 1B). Although patients were managed by a mixture of adult physicians and paediatricians, the overall therapeutic strategy was consistent, with the use of a common intensified protocol for the poor risk patients identified either clinically or by the index. We present the results of this treatment strategy.

## PATIENTS AND METHODS

The Northern Region Lymphoma Group (NRLG) collected data prospectively on all patients diagnosed with HL in the Northern region of England, as previously described (Taylor *et al*, 1998; Proctor *et al*, 2002). In addition, the Northern Region Young Person's Malignancy Registry (NRYPMR) collects data at presentation from all patients, aged ⩽25 years diagnosed with a malignant disease in the same geographical area (Cotterill *et al*, 2000). Patients were included in the present study if they presented between 1 January 1991 and 31 December 2000 with histologically confirmed, newly diagnosed HL, were aged 13–19 years (inclusive) at diagnosis and were normally resident in the area at the time of diagnosis. Histological material from all patients was centrally reviewed prior to patient inclusion on the register as previously described (Taylor *et al*, 1998).

### Registration and follow-up

The following data were collected from each patient at the time of registration: age, sex, histological subtype, full blood count, clinical stage at presentation, presence or absence of bulk disease, presence or absence of B-symptoms and first-line treatment modality. Level of haemoglobin, absolute lymphocyte count, age, stage and bulk disease assessment were required to calculate the SNLG index (Proctor *et al*, 1991).

Follow-up data regarding attainment of remission, relapse, requirement for second-line and subsequent therapy, date of death and cause of death were obtained from treating physicians or case-note review. Follow-up was to June 2005, or the date that the patient was last known to be alive. Data collection and analysis were undertaken with approval of the local ethical committee.

### Treatment strategy

Patients aged 16–19 years were managed using a risk-adapted strategy based on the SNLG prognostic index (Proctor *et al*, 1991).

#### High-risk patients

Patients identified as high risk were eligible for the SNLG-HD III trial protocol (Proctor *et al*, 2002), which involved treatment with three consecutive 28-day cycles of an eight-drug regimen (PVACEBOP: procarbazine, vinblastine, doxorubicin, chlorambucil, etoposide, bleomycin, vincristine and prednisolone; Table 1a). This was followed by involved-field radiotherapy (RT) to residual masses or sites of original bulk disease. Patients who gained a complete remission (CR) or good partial remission (GPR) were then randomised between two further cycles of PVACEBOP chemotherapy, or an autologous haemopoietic stem cell transplant (HSCT) with etoposide and melphalan preconditioning. High-risk patients who did not consent to trial entry were allowed to have this chemotherapy treatment off-study. Patients who relapsed or failed to remit were treated with the salvage chemotherapy regimen IVE (ifosfamide, etoposide (VP16) and epirubicin; Table 1b) and an autologous HSCT. After PVACEBOP, mobilisation of stem cells can be problematic if five courses have been used; so marrow harvest will be required for autograft in such circumstances. Relapse after PVACEPOP in this cohort was a rare event, so this was not a practical problem.

#### Standard risk patients

Patients with SNLG index <0.5 were treated according to Northern Region Haematology Guidelines. Early stage 1A and 2A patients received three courses of ChlVPP (chlorambucil, vinblastine, procarbazine and prednisolone) or ABVD (doxorubicin, bleomycin, vinblastine and dacarbazine) and involved-field RT to sites of residual or initial bulk disease. Before 1994, extended-field RT was used alone as primary therapy. Patients with higher-stage disease were treated with a minimum of six courses of a four-drug schedule (ChlVPP or ABVD) and involved-field RT.

### Survival analysis

Since (NLPHL) is now regarded as a subtype of low-grade non-HL, patients with this disease subtype have been analysed separately. Overall survival (OS) was measured from the date of diagnosis until the date of death. Disease-specific survival (DSS) was measured after censoring the data for deaths unrelated to HL. Event-free survival (EFS) was calculated from date of diagnosis to failure to achieve remission, relapse, disease progression, death or last follow-up.

### Statistical analysis

Prism 4.0 (GRAPHPAD Software, San Diego, CA, USA) was used for data analysis. Actuarial survival curves were compiled using the Kaplan–Meier method (Kaplan and Meier, 1958). The log-rank test was used to compare curves. Fisher's exact test was used where appropriate.

## RESULTS

### Patient characteristics

The former Northern Region of England has a population of 3.09 million. Over the 10 years of the study, 676 individuals presented with histologically confirmed HL, of whom 55 (8%) were aged 13–19 years at diagnosis. Of these, 48 patients (27 males, 21 females) had classical HL (cHL) and seven (six males, one female) had NLPHL. Median follow-up was 107 months (range 32–165) for surviving patients. The clinical characteristics of the patients and their outcomes during follow-up are shown in Tables 2a and b.

### Classical HL (cHL)

#### Therapy and outcome

Of the 48 patients in this group, 36 were aged ⩾16 years and were thus eligible for assessment using the SNLG index, and 12 were aged <16 years; application of the SNLG was not valid for this group. Outcomes by therapeutic modality and age are shown in Figure 2. In all, 46 of 48 patients (96%) achieved a remission after first-line therapy. Both primary refractory patients died of HL despite the use of high-dose salvage chemotherapy. Seven patients relapsed after primary therapy, three of these patients received radiotherapy alone as first-line treatment. All patients re-entered remission after salvage chemotherapy, but one subsequently died of HL. Two patients died of causes unrelated to HL; one died in a road traffic accident (RTA) and one (who also had cystic fibrosis) died of disseminated varicella zoster infection 96 months after presentation while in first CR. The 5-year and 10-year OS for this cohort was 93 and 86%, respectively (Figure 3A). DSS rates were 95 and 92% (Figure 3B).

Twenty-one patients, regarded as having high-risk disease, were treated using the PVACEBOP regimen (Table 1a). Of these, five patients received chemotherapy alone, 16 received combined therapy and four had an autologous transplantation in CR1 as part of the SNLG-HD III trial. With respect to SNLG prognostic index, 19 patients were aged ⩾16 years and 17 of 19 had an SNLG index ⩾0.5, thus reaching the criteria for high-risk disease. Two other patients with SNLG scores <0.5 were treated using the high-risk protocol at the discretion of the treating physician. Both are well in first CR and have experienced no late effects to date. Two patients, aged 13 and 15 years, were also treated using this protocol because of clinical poor risk profile with massive mediastinal disease. There were two primary refractory patients, who subsequently died of their disease, and one patient with a high-SNLG index entered CR but relapsed on several occasions, and died of progressive disease despite a reduced-intensity sibling HSCT.

Nineteen patients were treated using four-drug regimens (Figure 2). Combined chemotherapy and radiotherapy was used as first-line therapy in 11 cases, and chemotherapy was used alone in eight cases. All patients achieved CR. All patients treated in this group were considered to have standard risk disease, with one exception; a patient with a high-SNLG score was treated using this protocol, as she could not tolerate oral medication. She relapsed 12 months after diagnosis and was salvaged with IVE followed by an autologous HSCT. Another patient relapsed but was also alive in CR2 after salvage chemotherapy and an autologous HSCT. This patient's primary therapy was considered suboptimal as she became pregnant after two courses of chemotherapy and deferred further treatment until the postnatal period.

Eight patients with localised disease were managed with radiotherapy alone as first line-therapy (Figure 2). Five of these patients remained in CR1, three patients relapsed but had been salvaged with chemotherapy and were alive in CR. One patient, who also had cystic fibrosis, died 96 months after presentation of disseminated varicella zoster infection.

#### Compliance with regional protocol

As discussed, the SNLG index can only be reliably applied to patients aged ⩾16 years. With regard to these patients, regional guidelines were followed in 32 out of 36 cases. Two low-indexed patients were treated on the PVACEBOP protocol, one high-indexed patient received ABVD as she could not tolerate oral medication and one high indexed patient received radiotherapy alone.

#### Comparison of SNLG and Smith prognostic indices

**Figure 1:**
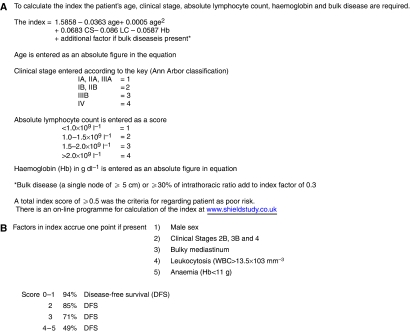
(**A**) Scottish and Newcastle Lymphoma Group prognostic index for Hodgkin's disease risk evaluation. (**B**) Prognostic index for childhood HD risk evaluation (Smith *et al*, 2003).

**Figure 2:**
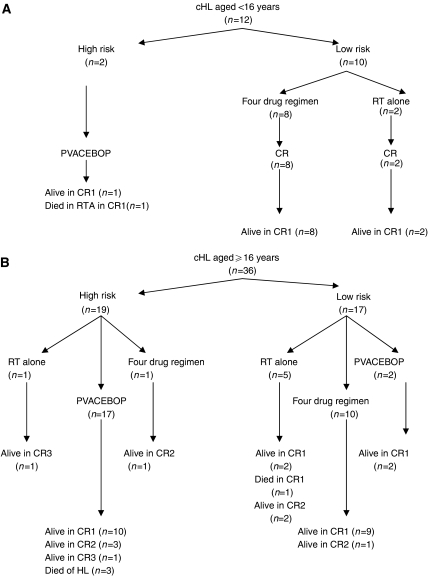
(**A**) Classical HL aged <16 years. (**B**) Classical HL aged ⩾16 years.

**Figure 3:**
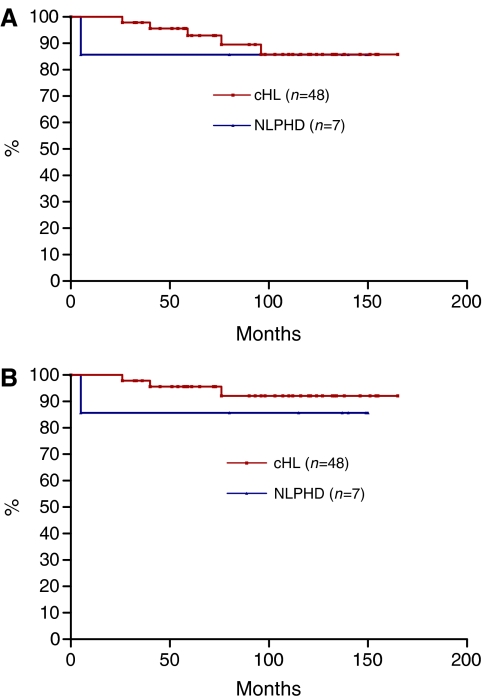
(**A**) Overall survival. (**B**) Disease-specific survival (censored for deaths unrelated to HL).

The Smith Index (Figure 1B), a prognostic score for childhood HL, was published in 2003 (Smith *et al*, 2003). It was therefore not available during the period these patients were treated. Nevertheless there is high concordance of Smith scores 3–5 as SNLG high-risk and Smith scores <3 as low-risk SNLG . Overall, there is agreement in risk allocation for 31 out of 36 patients. Five individuals were scored as high risk using the SNLG index (Figure 1A) and low risk using the Smith score. Of these patients, one received ABVD due to intolerance of oral medication and relapsed but received successful salvage therapy. Four patients received PVACEBOP-based therapy; two patients relapsed, one of whom had subsequently died of progressive disease. The Hasencleaver index could not be used in this cohort, as the serum albumin estimations had been recorded only in a minority of the cohort.

### NLPHL

This group comprised seven patients, three aged <16 years and four aged ⩾16 years. Therapy comprised radiotherapy alone (*n*=3), ChlVPP/ABVD+radiotherapy (*n*=2), ABVD+radiotherapy (*n*=1) and PVACEBOP+radiotherapy (*n*=1). Remission was achieved in 6/7 cases. The refractory patient was treated with PVACEBOP, achieved a partial remission but relapsed 4 months later with high-grade non-HL, and died of progressive disease 5 months after diagnosis. Two other patients relapsed but were salvaged with chemotherapy and autologous HSCT.

## DISCUSSION

Hodgkin's lymphoma in patients aged over 15 years was a subject of substantial research interest in the former Northern Region UK from 1982 onwards. This led to the development of the SNLG prognostic index in 1985, and although not published until 1991, it was used prospectively to define risk status as ‘standard’ or ‘high’ from 1987 (Proctor *et al*, 1991). It became the defining element of the SNLG-HD III randomised study for high-risk patients, which ran from 1988 to 1998 (Proctor *et al*, 2002). As a result, adolescents in the region who developed HL were prospectively assessed as described above, hence the early emergence of the risk – related strategy for the over 16 years cohort described here.

For patients with cHL, the 5-year and 10-year OS for this cohort was 93 and 86%, respectively. Overall survival for patients with early stage (stages IA, IIA) was 100 and 91%, respectively. These results compare favourably with other published studies, which have included adolescent patients (Table 3).

It should be borne in mind that, with the exception of the present study and those of Yung *et al* (2004) and Foltz *et al* (2006), all of the studies in Table 3 included younger children in addition to adolescents. It is not clear whether the prognosis of HL is the same in young children and teenagers; younger children have a better outcome than adolescents in some (Weiner *et al*, 1991) but not all studies (Schellong *et al*, 1999). In their study, specifically investigating adolescents with HL, Yung *et al* (2004) demonstrated a 5-year OS of 81% and EFS of 50% in a cohort of 210 patients. These patients, aged 15–17 years, were diagnosed from 1970–1997 and were treated according to adult protocols. Based on these data, the authors postulated that adolescents may be best treated using paediatric rather than adult treatment protocols. Survival data presented in our own study, using an adult risk-related approach, are better than those reported by Yung and co-workers. There are clearly several differences between the study of Yung and co-workers and the present study. These need to be considered when comparing results. The present study is population-based, the age range of the patients treated is broader and the patients were treated more recently than those described by Yung and co-workers. There was, however, no difference in survival by decade of treatment in the latter study. The present study describes the use of a risk-adapted treatment strategy and has demonstrated outcomes, which are comparable with those published by a number of paediatric groups (Ekert *et al*, 1988; Shankar *et al*, 1998; Schellong *et al*, 1999; Landmann-Parker *et al*, 2000; Smith *et al*, 2003; Oguz *et al*, 2005).

Recently Foltz *et al* (2006) have published the population experience of the British Columbia Cancer Agency lymphoid cancer database. The group included 259 individuals aged 16–21 yrs treated with adult therapy protocols. Overall survival and progression-free survival at 10 years were 91 and 77%, respectively. These survival data are similar to our own, with 10-year OS and EFS of 86 and 78% respectively, and suggest that adolescents can do well when managed using adult protocols.

The possible impact of the population-based nature of the data collected in this series and that of Foltz and co-workers is worthy of consideration. Among older patients with HL, it has been repeatedly demonstrated that the survival data in population-based studies are poorer than those from clinical trials (Kennedy *et al*, 1985; Appleton *et al*, 1995; Clarke *et al*, 2001; Proctor *et al*, 2002; Jarrett *et al*, 2005). This effect is likely to be due, at least in part, to the exclusion of frailer elderly individuals from clinical trials. It is not clear, however, whether a population-based approach may have a different impact in adolescent patients. It is conceivable that young patients with HL and poor prognostic features may enter trials in preference to those with lower-risk disease and indeed many clinical trials focus on those patients with poorer-risk disease (Hudson *et al*, 1993, 2004; Hutchinson *et al*, 1998; Friedmann *et al*, 2002). This may be another explanation of the differences between the present investigation and that of Yung co-workers; most of the patients in the latter study were registered at the time of recruitment to clinical trials.

While several studies (Jenkin *et al*, 1982; Shankar *et al*, 1998; Schellong *et al*, 1999; Oguz *et al*, 2005; Foltz *et al*, 2006) stratified patients to receive therapy based on stage of disease at presentation, no group specifically took into account additional prognostic factors such as those that comprise the SNLG index. Given the population-based nature of our study and the age range of patients included, we contend that the survival data presented in this cohort, the small number of patients requiring salvage therapy, and to date lack of secondary malignancy justifies our risk-adapted approach. Using an alternative strategy, Landmann-Parker *et al* (2000), stratified patients based on their response to an initial four cycles of chemotherapy. This group studied only patients with early stage disease; the results are similar to our own for patients with IA and IIA disease.

Current and proposed trials are assessing the use of PET scanning during therapy to inform the risk issue on an individual patient during treatment. This approach seems to have much merit as it allows standard or escalated therapy to be introduced according to response. The additional value of such an approach is that fertility might be better preserved in male patients using ABVD alone. Certainly eight-drug schedules, such as PVACEBOP, are associated with inevitable male sterility, although female fertility in the under 40 years patients on SNLG-HD III (Proctor *et al*, 2002) has been well preserved. We would argue that the new study approaches should be linked to assessment of existing prognostic indices to further assess their role in defining risk.

Nodular lymphocyte-predominant HL is now recognised as being a form of low-grade B-cell non-HL rather than a variant of cHL (Stoler *et al*, 1995; Harris *et al*, 2000). Several studies, including the small cohort investigated in the present series, have failed to demonstrate a survival difference between patients with NLPHL and those with other forms of the disease (Proctor *et al*, 1991; Weiner *et al*, 1991; Hunger *et al*, 1994). It seems unlikely that inclusion or exclusion of this small proportion of patients from the studies outlined in Table 3 would have a significant impact on overall results.

One of the other major considerations in the treatment of young patients with HL is the risk of late adverse effects of treatment. In the present study, follow-up is too short to allow an accurate assessment of the late effects of this risk-adapted therapeutic approach, but the results are generally encouraging; no secondary leukaemia or solid tumours have been reported in this cohort to date.

## CONCLUSION

This study demonstrates that a coordinated approach, involving adult and paediatric physicians, and the use of a risk-adapted treatment protocol are effective in the management of HL in adolescents. Given the relative rarity of HL in adolescents, a national population-based registry approach could provide valuable information with respect to best practice in addition to providing a vehicle to promote recruitment into clinical trials.

## Figures and Tables

**Table 1:** PVACE BOP primary therapy for poor-risk cases (SNLG index >0.5)

(*a*) *For high-risk Hodgkin's disease*	
Day 1	Vinblastine (6 mgm^−2^ IV)
Day 1–3	Etoposide (IV 100 mgm^−2^ × 1 dose)
	Oral (200 mgm^−2^ × 2 doses)
Days 1–14	Procarbazine (100 mgm^−2^ oral)
Days 1–14	Chlorambucil (6 mgm^−2^ oral)
Day 8	Adriamycin (25 mgm^−2^ IV)
Day 8	Vincristine (2 mg IV)
Day 15	Bleomycin (6 mgm^−2^ IV)
Day 22	Bleomycin (6 mgm^−2^ IV)
Days 14–28	Prednisolone (40 mg daily oral)
Day 29	=Day 1 of next course
	
*(b) Ifosphamide, VP16 and epirubicin (IVE) for relapsed or progressive Hodgkin's disease*	
VP16	200 mgm^−2^day^−1^ as 2 h infusion days 1–3
Epirubicin	50 mgm^−2^day^−1^ IV day 1 (bolus)
Ifosphamide	3 gm^−2^ 24 h infusions days 1–3 with MESNA cover in 2.5 l dextrose saline

Abbreviations: IV, intravenously; IVE, ifosfamide, etoposide (VP16) and epirubicin; PVACEBOP, procarbazine, vinblastine, doxorubicin, chlorambucil, etoposide, bleomycin, vincristine and prednisolone; SNLG, Scottish and Newcastle Lymphoma Group.

Bleomycin omitted from cycles 4 and 5 if patients have had mantle/mediastinal radiotherapy.

A 100 mg hydrocortisone IV administered with bleomycin.

Septrin (960 mg), once daily, should be given throughout treatment on Mondays, Wednesdays and Fridays.

On day 1 of each cycle before ifosphamide is administered a loading dose of 1.8 gm^−2^ of MESNA is given as an IV bolus.

A final infusion of MESNA 5.4 g/m^2^ (60% of total ifosphamide dose) will be given in 1.5 l of dextrose saline given over 12 h.

Three cycles, at 21-day intervals (neutrophils >1.5 × 10/9/l and platelets >75 × 10/9/l) for a total of three courses.

Patients receive phenytoin (po 300 mg) daily from days 1–5.

**Table 2:** Clinical features and outcome of the cohort

	**Gender**		**Clinical stage**								**Pathology**		**SNLG index**		**Smith index**				
**Age**	**M/F**	**IA**	**IB**	**IIA**	**IIB**	**IIIA**	**IIIB**	**IVA**	**IVB**	**NS**	**MC**	**<0.5**	**⩾0.5**	**0/1**	**2**	**3**	**4/5**	**5 year DSS**	**5 year EFS**
*(a) Classical HL*																			
<16 years	8/4	1	1	5	0	1	1	0	3	1	11	N/A	N/A	4	3	4	1	100%	90%
**⩾**16 years	19/17	3	0	10	5	4	7	2	5	6	30	17	19	15	8	7	6	94%	79%
																			
**Gender**		**Clinical stage**								**SNLG index**			**Smith index**						
**M/F**	**IA**	**IB**	**IIA**	**IIB**	**IIIA**	**IIIB**	**IVA**	**IVB**	**N/A**	**<0.5**	**⩾0.5**	**0/1**	**2**	**3**	**4/5**	**5 year DSS**	**5 year EFS**		
*(b) Lymphocyte-predominant HL*																			
6/1	2	1	1	0	3	0	0	0	3	3	1	6	1	0	0	86%	67%		

Abbreviations: DSS, disease-specific survival; EFS, event-free survival; HL, Hodgkin's lymphoma; SNLG, Scottish and Newcastle Lymphoma Group.

**Table 3:** Published outcomes for adolescent patients with HL

**Authors**	**Publication year**	**Number of patients**	**Ages (years)**	**Median age (years)**	**Stages**	**NLPHL included**	**Median F/U (month)**	**CR rate**	**5 year OS**	**5 year EFS**	**Study type**
Clarke *et al*	2001	5630	15–44	NS	NS	Yes	53	NS	NS	5 year DFS 90%	Retrospective, population based
Donaldson and Link	1987	55	1.5–15	10	All	Yes	90	93%	89% at 15 years	5 year RFS 90%	Prospective, observational
Donaldson *et al*	2002	110	3–20	13	All	Yes	67	100%	99%	93%	Prospective, observational, focused on LR disease
Ekert *et al*	1988	53	3–16	10	All	Yes	45	96%	94%	92%	Prospective observational
Foltz *et al*	2006	259	16–21	19	All	Yes	102	97%	91%	10 year PFS 77%	Prospective observational
Friedmann *et al*	2002	56	8–18	15	All (only HR I–II)	Yes	108	94%	82%	68%	Prospective, observational, focused on HR disease
Hudson *et al*	1993	85	4–20	14	IIA–IVB	Yes	49	98%	93%	5 year DFS 93%	Prospective, observational, focused on HR disease
Hudson *et al*	2004	159	2–19	15	All (only HR I–II)	Yes	70	NS	93%	76%	Prospective, observational, focused on HR disease
Hunger *et al*	1994	57	<18	12	All	Yes	80	100%	96%	93%	Prospective, observational
Hutchinson *et al*	1998	111	<21	NS	III/IV only	Yes	74	NS	87% at 4 years	82% at 4 years	RCT, focused on HR disease
Jenkin *et al*	1982	110	<16	NS	All	Yes	70	NS	92%	RFS 68%	Prospective, observational
Jones *et al*	Present paper 2007	55	13–19	16	All	Yes	107	93%	91%	NS	Prospective, population-based
Landman-Parker *et al*	2000	202	3–18	12	I/II only	No	74	NS	98%	91%	Prospective, observational, risk-adapted approach
Nachman *et al*	2002	829	<21	NS	All	Yes	NA	83%	95% at 3 years	87% at 3 years	RCT for patients in CR after chemotherapy
Oguz *et al*	2005	65	2–15	7	All	Yes	73	95%	96%	91%	Prospective, observational
Schellong *et al*	1999	578	2–17	13	All	Yes	61	NS	98%	91%	Prospective, observational
Shanker *et al*	1998	54	2–19	10	All	Yes	66	76%	Stage I–III 93% Stage IV 44%		Prospective, observational
Smith *et al*	2003	328	2–20	14	All	Yes	59	NS	93%	DFS at 5 years 83%	Prospective, observational using risk-adapted protocols
Weiner *et al*	1997	179	4–20	13	**⩾**IIB	Yes	NS	90%	92%	79%	RCT focused on HR disease
Yung *et al*	2004	210	15–17	16	All	Yes	199	76%	81%	50%	Retrospective, observational

Abbreviations: CR, complete remission; DFS, disease-free survival; EFS, event-free survival; HL, Hodgkin's lymphoma; HR, high risk; LR, low risk. NA, not available; NLPHL, nodular lymphocyte-predominant Hodgkin's lymphoma; NS, non-significant; OS, overall survival; RCT, randomised controlled trial.
